# Supplementary material for: HIVprotI: an integrated web based platform for prediction and design of HIV proteins inhibitors
Source: J Cheminform. 2018 Mar 9;10:12. doi: 10.1186/s13321-018-0266-y (PMC5845081; doi:10.1186/s13321-018-0266-y)
Supplement: Supplementary file 2 — Additional file 2. Source code of HIVProtI web server. [file 13321_2018_266_MOESM2_ESM.zip › HIVprotI_Source-code/hivprotI/weka-3-6-6/doc/weka/filters/unsupervised/attribute/NominalToBinary.html]

NominalToBinary


---


|  |  |  |
| --- | --- | --- |
| **Overview** \| **Package** \| **Class** \| **Tree** \| **Deprecated** \| **Index** \| **Help** \| **Weka's home** | |  |
| **PREV CLASS**   **NEXT CLASS** | **FRAMES**    **NO FRAMES**     **All Classes** |  |
| SUMMARY: NESTED \| FIELD \| CONSTR \| METHOD | DETAIL: FIELD \| CONSTR \| METHOD |  |


---


## weka.filters.unsupervised.attribute Class NominalToBinary

```
java.lang.Object
  weka.filters.Filter
      weka.filters.unsupervised.attribute.NominalToBinary
```

**All Implemented Interfaces:**: java.io.Serializable, CapabilitiesHandler, OptionHandler, RevisionHandler, UnsupervisedFilter

---

``` public class NominalToBinary extends Filter implements UnsupervisedFilter, OptionHandler ```

Converts all nominal attributes into binary numeric attributes. An attribute with k values is transformed into k binary attributes if the class is nominal (using the one-attribute-per-value approach). Binary attributes are left binary, if option '-A' is not given.If the class is numeric, you might want to use the supervised version of this filter.


Valid options are:

```
 -N
  Sets if binary attributes are to be coded as nominal ones.
```

```
 -A
  For each nominal value a new attribute is created, 
  not only if there are more than 2 values.
```

```
 -R <col1,col2-col4,...>
  Specifies list of columns to act on. First and last are 
  valid indexes.
  (default: first-last)
```

```
 -V
  Invert matching sense of column indexes.
```

**Version:**
:   $Revision: 5543 $

**Author:**
:   Eibe Frank (eibe@cs.waikato.ac.nz)

**See Also:**: Serialized Form

---

**Constructor Summary**

`NominalToBinary()`
  
          Constructor - initialises the filter


| **Method Summary** | |
| --- | --- |
| `java.lang.String` | `attributeIndicesTipText()`             Returns the tip text for this property |
| `java.lang.String` | `binaryAttributesNominalTipText()`             Returns the tip text for this property |
| `java.lang.String` | `getAttributeIndices()`             Gets the current range selection |
| `boolean` | `getBinaryAttributesNominal()`             Gets if binary attributes are to be treated as nominal ones. |
| `Capabilities` | `getCapabilities()`             Returns the Capabilities of this filter. |
| `boolean` | `getInvertSelection()`             Gets whether the supplied columns are to be removed or kept |
| `java.lang.String[]` | `getOptions()`             Gets the current settings of the filter. |
| `java.lang.String` | `getRevision()`             Returns the revision string. |
| `boolean` | `getTransformAllValues()`             Gets if all nominal values are turned into new attributes, not only if there are more than 2. |
| `java.lang.String` | `globalInfo()`             Returns a string describing this filter |
| `boolean` | `input(Instance instance)`             Input an instance for filtering. |
| `java.lang.String` | `invertSelectionTipText()`             Returns the tip text for this property |
| `java.util.Enumeration` | `listOptions()`             Returns an enumeration describing the available options. |
| `static void` | `main(java.lang.String[] argv)`             Main method for testing this class. |
| `void` | `setAttributeIndices(java.lang.String rangeList)`             Sets which attributes are to be acted on. |
| `void` | `setBinaryAttributesNominal(boolean bool)`             Sets if binary attributes are to be treates as nominal ones. |
| `boolean` | `setInputFormat(Instances instanceInfo)`             Sets the format of the input instances. |
| `void` | `setInvertSelection(boolean invert)`             Sets whether selected columns should be removed or kept. |
| `void` | `setOptions(java.lang.String[] options)`             Parses a given list of options. |
| `void` | `setTransformAllValues(boolean bool)`             Sets whether all nominal values are transformed into new attributes, not just if there are more than 2. |
| `java.lang.String` | `transformAllValuesTipText()`             Returns the tip text for this property |

**Methods inherited from class weka.filters.Filter**

`batchFilterFile, batchFinished, filterFile, getCapabilities, getOutputFormat, isFirstBatchDone, isNewBatch, isOutputFormatDefined, makeCopies, makeCopy, numPendingOutput, output, outputPeek, toString, useFilter, wekaStaticWrapper`

**Methods inherited from class java.lang.Object**

`equals, getClass, hashCode, notify, notifyAll, wait, wait, wait`

**Constructor Detail**

### NominalToBinary

```
public NominalToBinary()
```

:   Constructor - initialises the filter


**Method Detail**

### globalInfo

```
public java.lang.String globalInfo()
```

:   Returns a string describing this filter

    :   **Returns:**: a description of the filter suitable for displaying in the explorer/experimenter gui

---


### getCapabilities

```
public Capabilities getCapabilities()
```

:   Returns the Capabilities of this filter.

    :   **Specified by:**: `getCapabilities` in interface `CapabilitiesHandler` **Overrides:**: `getCapabilities` in class `Filter`
    :   **Returns:**: the capabilities of this object **See Also:**: `Capabilities`

---


### setInputFormat

```
public boolean setInputFormat(Instances instanceInfo)
                       throws java.lang.Exception
```

:   Sets the format of the input instances.

    :   **Overrides:**: `setInputFormat` in class `Filter`
    :   **Parameters:**: `instanceInfo` - an Instances object containing the input instance structure (any instances contained in the object are ignored - only the structure is required). **Returns:**: true if the outputFormat may be collected immediately **Throws:**: `java.lang.Exception` - if the input format can't be set successfully

---


### input

```
public boolean input(Instance instance)
```

:   Input an instance for filtering. Filter requires all
    training instances be read before producing output.

    :   **Overrides:**: `input` in class `Filter`
    :   **Parameters:**: `instance` - the input instance **Returns:**: true if the filtered instance may now be collected with output(). **Throws:**: `java.lang.IllegalStateException` - if no input format has been set

---


### listOptions

```
public java.util.Enumeration listOptions()
```

:   Returns an enumeration describing the available options.

    :   **Specified by:**: `listOptions` in interface `OptionHandler`
    :   **Returns:**: an enumeration of all the available options.

---


### setOptions

```
public void setOptions(java.lang.String[] options)
                throws java.lang.Exception
```

:   Parses a given list of options.


    Valid options are:

    ```
     -N
      Sets if binary attributes are to be coded as nominal ones.
    ```

    ```
     -A
      For each nominal value a new attribute is created, 
      not only if there are more than 2 values.
    ```

    ```
     -R <col1,col2-col4,...>
      Specifies list of columns to act on. First and last are 
      valid indexes.
      (default: first-last)
    ```

    ```
     -V
      Invert matching sense of column indexes.
    ```

    :   **Specified by:**: `setOptions` in interface `OptionHandler`
    :   **Parameters:**: `options` - the list of options as an array of strings **Throws:**: `java.lang.Exception` - if an option is not supported

---


### getOptions

```
public java.lang.String[] getOptions()
```

:   Gets the current settings of the filter.

    :   **Specified by:**: `getOptions` in interface `OptionHandler`
    :   **Returns:**: an array of strings suitable for passing to setOptions

---


### binaryAttributesNominalTipText

```
public java.lang.String binaryAttributesNominalTipText()
```

:   Returns the tip text for this property

    :   **Returns:**: tip text for this property suitable for displaying in the explorer/experimenter gui

---


### getBinaryAttributesNominal

```
public boolean getBinaryAttributesNominal()
```

:   Gets if binary attributes are to be treated as nominal ones.

    :   **Returns:**: true if binary attributes are to be treated as nominal ones

---


### setBinaryAttributesNominal

```
public void setBinaryAttributesNominal(boolean bool)
```

:   Sets if binary attributes are to be treates as nominal ones.

    :   **Parameters:**: `bool` - true if binary attributes are to be treated as nominal ones

---


### transformAllValuesTipText

```
public java.lang.String transformAllValuesTipText()
```

:   Returns the tip text for this property

    :   **Returns:**: tip text for this property suitable for displaying in the explorer/experimenter gui

---


### getTransformAllValues

```
public boolean getTransformAllValues()
```

:   Gets if all nominal values are turned into new attributes, not only if
    there are more than 2.

    :   **Returns:**: true all nominal values are transformed into new attributes

---


### setTransformAllValues

```
public void setTransformAllValues(boolean bool)
```

:   Sets whether all nominal values are transformed into new attributes, not
    just if there are more than 2.

    :   **Parameters:**: `bool` - true if all nominal value are transformed into new attributes

---


### invertSelectionTipText

```
public java.lang.String invertSelectionTipText()
```

:   Returns the tip text for this property

    :   **Returns:**: tip text for this property suitable for displaying in the explorer/experimenter gui

---


### getInvertSelection

```
public boolean getInvertSelection()
```

:   Gets whether the supplied columns are to be removed or kept

    :   **Returns:**: true if the supplied columns will be kept

---


### setInvertSelection

```
public void setInvertSelection(boolean invert)
```

:   Sets whether selected columns should be removed or kept. If true the
    selected columns are kept and unselected columns are deleted. If false
    selected columns are deleted and unselected columns are kept.

    :   **Parameters:**: `invert` - the new invert setting

---


### attributeIndicesTipText

```
public java.lang.String attributeIndicesTipText()
```

:   Returns the tip text for this property

    :   **Returns:**: tip text for this property suitable for displaying in the explorer/experimenter gui

---


### getAttributeIndices

```
public java.lang.String getAttributeIndices()
```

:   Gets the current range selection

    :   **Returns:**: a string containing a comma separated list of ranges

---


### setAttributeIndices

```
public void setAttributeIndices(java.lang.String rangeList)
```

:   Sets which attributes are to be acted on.

    :   **Parameters:**: `rangeList` - a string representing the list of attributes. Since the string will typically come from a user, attributes are indexed from 1. eg: first-3,5,6-last **Throws:**: `java.lang.IllegalArgumentException` - if an invalid range list is supplied

---


### getRevision

```
public java.lang.String getRevision()
```

:   Returns the revision string.

    :   **Specified by:**: `getRevision` in interface `RevisionHandler` **Overrides:**: `getRevision` in class `Filter`
    :   **Returns:**: the revision

---


### main

```
public static void main(java.lang.String[] argv)
```

:   Main method for testing this class.

    :   **Parameters:**: `argv` - should contain arguments to the filter: use -h for help


---


|  |  |  |
| --- | --- | --- |
| **Overview** \| **Package** \| **Class** \| **Tree** \| **Deprecated** \| **Index** \| **Help** \| **Weka's home** | |  |
| **PREV CLASS**   **NEXT CLASS** | **FRAMES**    **NO FRAMES**     **All Classes** |  |
| SUMMARY: NESTED \| FIELD \| CONSTR \| METHOD | DETAIL: FIELD \| CONSTR \| METHOD |  |


---
